# Ontogeny of collective behaviour

**DOI:** 10.1098/rstb.2022.0065

**Published:** 2023-04-10

**Authors:** Isabella Benter Muratore, Simon Garnier

**Affiliations:** Department of Biological Sciences, New Jersey Institute of Technology, Newark, NJ 07102, USA

**Keywords:** collective architecture, ontogeny, development, social insects, collective behaviour, self-assemblage

## Abstract

During their lifetime, superorganisms, like unitary organisms, undergo transformations that change the machinery of their collective behaviour. Here, we suggest that these transformations are largely understudied and propose that more systematic research into the ontogeny of collective behaviours is needed if we hope to better understand the link between proximate behavioural mechanisms and the development of collective adaptive functions. In particular, certain social insects engage in self-assemblage, forming dynamic and physically connected architectures with striking similarities to developing multicellular organisms, making them good model systems for ontogenetic studies of collective behaviour. However, exhaustive time series and three-dimensional data are required to thoroughly characterize the different life stages of the collective structures and the transitions between these stages. The well-established fields of embryology and developmental biology offer practical tools and theoretical frameworks that could speed up the acquisition of new knowledge about the formation, development, maturity and dissolution of social insect self-assemblages and, by extension, other superorganismal behaviours. We hope that this review will encourage an expansion of the ontogenetic perspective in the field of collective behaviour and, in particular, in self-assemblage research, which has far-reaching applications in robotics, computer science and regenerative medicine.

This article is part of a discussion meeting issue ‘Collective behaviour through time’.

## 1. Introduction

Julian Huxley contended that there were ‘three major problems of Biology’ [1]: causation, survival value and evolution [2]. Niko Tinbergen, in his famous ‘On aims and methods of Ethology’ [1], replied to Huxley that there was in fact a fourth: ontogeny, that is, the problem of how biological mechanisms change over time. Tinbergen, however, admitted in the same essay that the ontogenetic problem in the context of behaviour was somewhat difficult to circumscribe and that its study had, as a result, a slow start in the then rapidly expanding and maturing field of ethology.

Since then, it is safe to say that the concerns of the 1973 Nobel Prize winner have been answered. The ontogeny of behaviour is a very prolific branch of the modern behavioural sciences [3], with the exception of the study of collective behaviour. Indeed, a significant portion of the collective behaviour literature is dedicated to the single form (or ‘synchronic’) perspective with about an equal amount of interest given to the proximate mechanisms of collective behaviour (e.g. the biological branch of the field of swarm intelligence [4]) and to their adaptive significance for the group as a whole (e.g. in eusocial species) or for its members individually. On the other hand, the historical (or ‘diachronic’) perspective is—arguably—dominated by evolutionary/phylogenetic studies, in particular, focused on the evolution of sociality in general [5], but also, to a lesser extent, of more specific forms of collective behaviour (e.g. schooling/flocking, nest architecture).

The ontogenetic question, however, appears to be largely ignored in the field of collective behaviour. It is widely studied in the context of individual social skills (e.g. social development and its associated disorders; acquisition of language and other forms of social transmission of information; etc. [6–8]) but rarely tackled explicitly in a group-level context. For instance group size—which is one of the strongest markers of a group's developmental stage, like body mass/size for an individual organism [9]—is typically represented as a fixed-value factor, or one with only a few values, in models of collective behaviours, and not as a quantity that varies continuously over time alongside and in interaction with the group's behaviour [10–12]. In other words, it is treated as an external environmental parameter despite being an intrinsic property of the group and a result of its historical trajectory. In this opinion review, we will argue that an ontogenetic approach which examines *transitions* in group size, composition and other collective features—and not just their direct effect on the group's behaviour—could round out the field of collective behaviour by promoting the development of longitudinal studies that investigate the entire life history of emergent group properties.

Ontogeny encompasses the understanding of how an organism forms, or its conception, and how it develops, matures and eventually decays and dies [1,13]. In particular, ontogenetic studies seek to identify the factors, whether physiological or environmental, that control the timing of and transitions between the different life stages of an organism. As such, the framework of ontogeny encourages a historical rather than static perspective on an organism's biology, and time takes priority over space when setting the scale of observation.

This framework is commonly applied to study the coordinated actions of dividing embryonic cells [14] and, therefore, its principles should be directly applicable to examine the formation of social groups or the progression of collective behaviours over time. Like embryonic processes, many collective animal behaviours generate emergent patterns exhibited at the level of the group as the result of interactions between its members [4,11]. They can be governed by simple behavioural rules that combine with environmental inputs to form complex results [4,15,16], and as such may not require sophisticated cognitive processing [17–23]. For example, when some groups of animals make movement decisions, they can do so without relying on signalling or knowledge of decisions made by peers [24,25]. Similarly, the complex and functional architectures of a developing organism are the result of self-organizing processes between cognitively limited units. Embryonic cells secrete and respond to growth factors and other proteins to self-assemble, or physically organize themselves without centralized control, into patterns comprising the tissues of a developing organism [26–28]. Similar to the swarms and other collective structures built by social organisms, embryonic cells assume detailed formations, such as the eye and its mosaically patterned microscopic structures [29] or the skeleton [30], without any central organization [31]. Eusocial insects also build intricate self-assemblages [32], and nests, relying on pheromonal instructions, environmental inputs and repetitive instinctual behaviours [33–35].

Unlike embryology, which considers how the individual cells change their behaviour over time allowing for transitions between different and often increasingly complex collective states, the field of collective animal behaviour tends to study these collective states in isolation. This is partly owing to the difficulty of tracking individuals and collectives over extended periods of time [36–38] and of artificially manipulating individual states and group dynamics. These practical limitations are, however, progressively vanishing as new observation technologies are being developed. In this context, the preponderance of literature on embryonic development offers promising avenues for the study of collective animal behaviour through time. Social groups have often been compared to multicellular organisms [39–43], and to augment this school of thought, this discussion will emphasize some of their features that can be compared to developing embryos, and the insights to be gained from the tools used to study multicellular ontogeny.

## 2. Collective ontogeny

**Figure 1.**
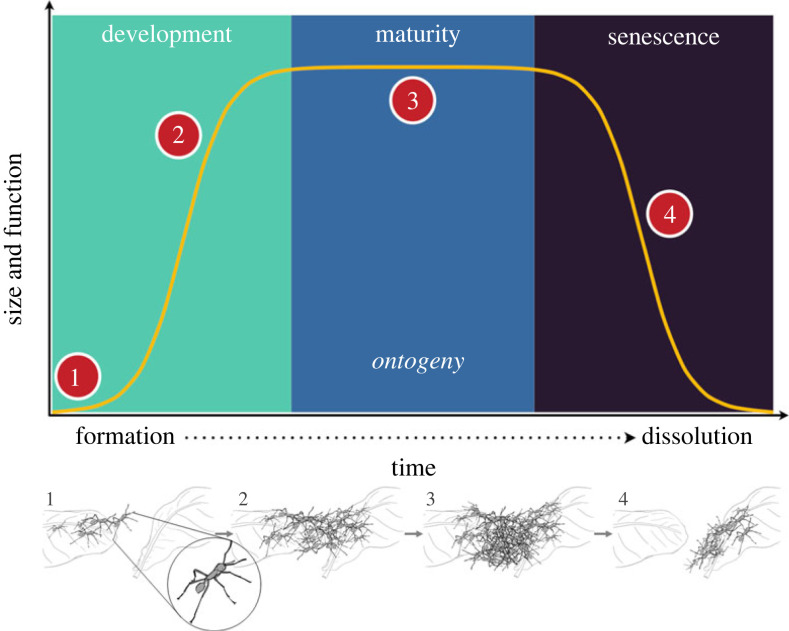
Schematic description of an ontogenetic trajectory, from the formation to the dissolution of a biological structure. In army ants, the ontogenetic trajectory of a support structure (e.g. bridge, scaffold) is controlled by a combination of environmental and social information. (1) The structure is initiated by substrate heterogeneities that impede the movement of the ants along their foraging trails and increase the probability of an ant anchoring itself into the substrate. (2) As the structure grows by the addition of ants attaching to it, the constraint on movement along the trail decreases, which reduces the probability of new ants joining the structure and leads to its progressive stabilization. (3) Adjustments to bridge length are buffered against minor, temporary fluctuations in obstacle topology by hysteresis. (4) Finally, when the traffic along the trail decreases significantly (e.g. at the end of a foraging bout), the decrease in tactile cues experienced by ants comprising the bridge leads them to leave at a faster rate, causing the dissolution of the bridge.

As with multicellular development, social groups undergo ontogenetic changes over their lifespan. These changes can be—generally speaking—organized into four successive life stages with somewhat blurry boundaries: (i) formation (or birth), (ii) growth and development, (iii) maturity (or adulthood) and finally, (iv) dissolution (or death) (figure 1). While these stages of ontogeny have been described extensively in a variety of unitary organisms, groups on the superorganism scale are not generally considered in this level of detail. Applying a similar naming scheme to different stages of the collective life cycle could attract more studies to each phase, and create more consistency across studies.

Moreover, ontogeny as a concept encompasses multiple scales of size and time, and more than one level may be considered at the same time. Indeed, it is possible to consider the ontogenetic trajectory of an entire organism but also of its components separately (e.g. a cell or group of cells). Similarly, collective ontogeny can be considered over the lifetime of a social group (e.g. from the emergence of a new ant queen to the death of the colony she founded) but also over the duration of a specific collective action, such as the construction/disassembly of an army ant bivouac, for instance. While our review prioritizes discussion of relatively short-lived collective behaviours and self-assemblages of multicellular individuals, which are more easily studied, the developmental viewpoint that we are advocating for can also be applied to the longer lifespans of nests and entire colonies.

### (a) Formation

**Figure 2.**
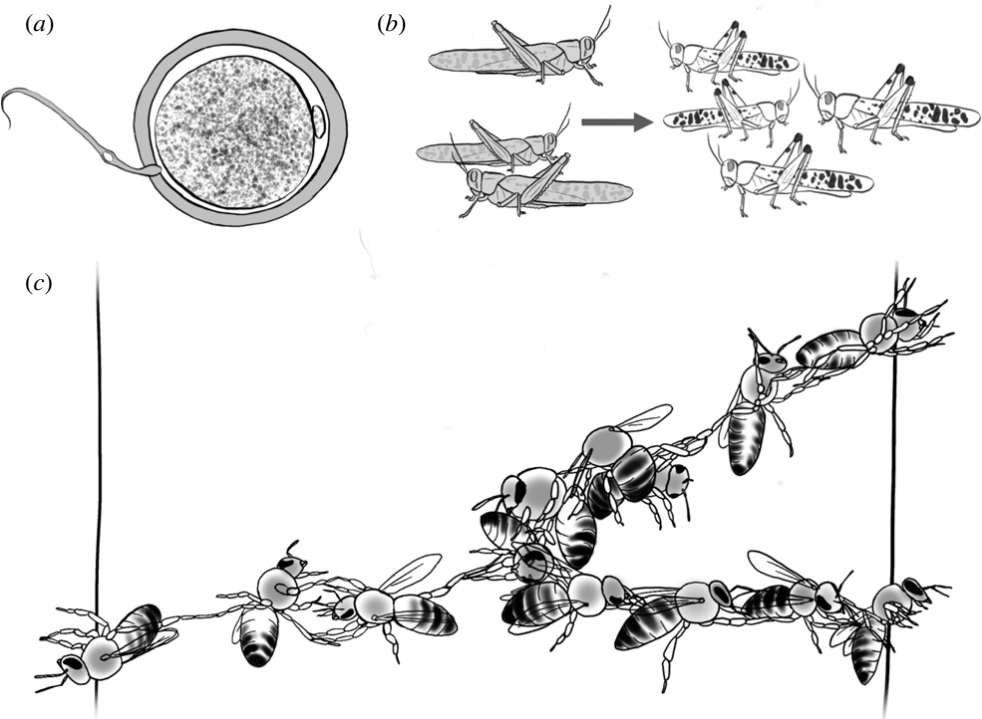
Fertilization (*a*), locust gregarization ((*b*); [44]), and the early stages of a honeybee festoon ((*c*); [45]) as examples of formation.

Fertilization, zygote formation and early cell divisions in embryos are well understood (figure 2*a*) [46–49]. However, for many transitory collective behaviours, factors leading to aggregation and the dynamics of the early stages of such aggregations, are not thoroughly characterized. In particular, terminology describing typical phases of formation is either unavailable or inconsistent across studies.

Even at levels of organization below embryos, enzymes and other organic molecules have been observed to self-assemble [50–52] and their relative chemical simplicity has enabled this process to be well understood and even modified or artificially induced [53,54]. Self-assembly also enables the behaviour of organelles and other cell components, for example, the highly plastic architecture of the cytoskeleton via the aggregation of actin filaments and tubulin dimers [55,56]. Such small-scale assemblages occur on timescales much faster than even those of transitory collective behaviours, as speed is needed for many processes on the organ/tissue scale such as the firing of neurons or fast-twitch muscles.

On a larger scale, the fertilization process and subsequent stages of zygote development are so well characterized that human embryos can be predictably preserved at an optimal state for survival and growth after implantation [57,58]. The same cannot be said for our ability to recognize or induce the early formation stages of social groups. However, some species offer greater insight into this question.

One system in particular in which the genesis of group formation is relatively well understood is the transition from the solitary state to the gregarious state in locusts (figure 2*b*) [59]. This can be triggered by tactile stimulation of the hindlegs acting as an indicator of a high density of individuals and leads locusts to undergo a hormonal change inducing social swarming behaviour [44]. This mechanosensory route of collective germination is now very well described and echoes the role of mechanical forces and hormonal pathways in influencing early embryo development [60].

Like locusts, blackworms alternate between solitary and group behaviour depending on the environment, congregating into mobile blobs when exposed to light and heat [61]. While not as detailed as our knowledge of locust gregarization, investigations of the specific cues causing this state change and the mechanical properties of the resulting blob enabled the construction of bioinspired robot aggregations [61], exemplifying the use of understanding group formation.

The initiation of most other collective behaviours is, however, not well studied in general. For instance, honeybees form ‘festoons’ of workers linked together in a lacework curtain formation (figure 2*c*) [62]. This form of transitory living architecture may be a precursor to the construction of the physical comb. However, the exact initiation of the comb-building process and how it relates to festooning is not yet fully understood [63].

In several ant species (e.g. army ants, fire ants, weaver ants), transitory collective behaviours may emerge in response to environmental factors similar to those influencing embryonic formation. For example, the location of temporary army ant nests—called bivouacs—is determined by local environmental conditions, in particular for the purpose of optimizing brood-rearing temperature [64–67]. However, in contrast to locust swarm formation and blackworm blob formation, we lack detailed descriptions of the morphology of nascent bivouacs or of the exact environmental and behavioural factors that cause ants to begin construction. This information is, however, known for other self-assemblages built by army ants, such as bridges and scaffolds, which are initiated in response to local constraints on ant movement along their foraging and migratory trails [68,69].

Knowing the factors that lead individuals to initiate specific types of collective behaviours could be instrumental in fields such as urban planning [70] and agriculture [71,72]. For example, the behaviour of locust swarms has a significant influence on global crop survival [73,74], while ant social behaviour has inspired a highly influential suite of algorithms that have, in turn, contributed to decisions about city planning [75–78]. Such knowledge could be augmented through careful tracking of individuals and data on social and environmental conditions prior to and leading up to collective actions. Hypotheses based on observed correlations with environmental and social factors (e.g. [79]) could then be used to artificially induce swarming until instigating factors are well characterized and initiate such behaviour robustly. Though challenges remain in implementing such artificial manipulations, some studies of hymenopteran behaviour have made use of simple techniques such as altering worker density or demography to successfully induce the formation of specific collective formations [80–85].

### (b) Development

**Figure 3.**
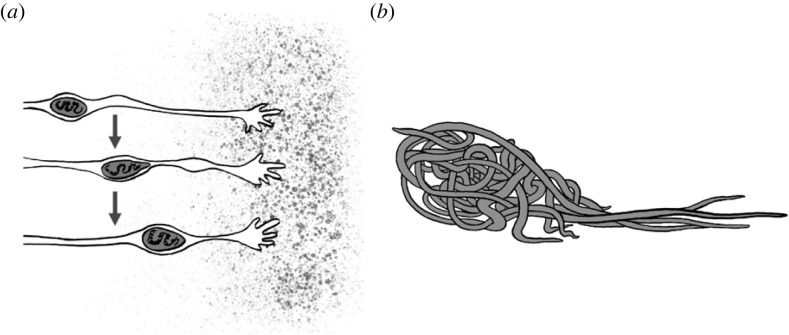
Neuron migration via chemotaxis ((*a*); [87]) and blackworm blob motility via division of labour ((*b*); [61]) as examples of development.

After their initiation, collective structures, like fertilized eggs, grow in size and develop functions (e.g. with respect to colony growth and demography [79]) until they reach a stable state; in other words, they undergo a form of embryogenesis. Stages of embryogenesis have been carefully described for over a century in humans and a variety of model systems [86], and function-building processes such as the chemotactic migration of neurons are well understood [87] (figure 3*a*). They are, however, often overlooked in studies of collective behaviours that tend to focus more often on the final, stable product of the group's actions. Nevertheless, the progressive transformations that the group undergoes on its way to its stable state can be key to understanding why a particular instance of collective behaviour is successful or not. For example, the exact sequence of individual choice events in the early stages of a collective decision is critical to determining whether the social feedback loop will lead to a beneficial collective outcome or not. Knowing the developmental trajectory of collective decisions—or any collective behaviour for that matter—may help explain why certain social feedback strategies have better success rates than others, or to design strategies that are more robust to developmental accidents.

Mechanical forces are among the best-known drivers of embryo development [88]. They are also prominent in many collective behaviours. However, the mechanical properties of self-assemblages and other collective behaviours and what physical conditions allow for their stable growth have only recently been studied in a preliminary fashion [89–91]. Given the shared features between collective behaviour and ontogeny highlighted earlier, we believe that there is great potential to better understand collective development and life cycles by focusing more attention on their physics.

For instance, honeybees may sense worker density inside their colony via the collisions they experience with other workers [81]. This mode of monitoring colony size triggers modifications of the workers' behaviours that can then lead to changes to comb structure [81]. Similarly, a recent study revealed that doryline army ants transition from group to mass raiding behaviour as their colony expands, without change to the rules of individual interactions [80]. *Temnothorax* workers are hypothesized to sense a quorum for group decision-making about nest relocation, which triggers them to switch from tandem running to carrying other ants to a new site, based on the rate at which they encounter peers at the site in question [83]. These examples suggest a very strong link between colony growth—and hence, the density of interactions—and functional development, similar to what can be observed in embryology.

Mechanical stresses are also likely to have a strong influence on both the development and final form of collective architectures. They have already been shown to shape the construction dynamics of small self-assemblages. For instance, the grippiness of the substrate on which army ants walk has a strong influence on their individual probability to build support structures to improve locomotion [69]. At low-grip locations, army ants will readily self-assemble into a scaffold but, as the scaffold grows in size and, therefore, improves the surface grip, subsequent ants will be less likely to join in the structure which will naturally halt its growth and development once it has become fully functional [69]. In larger self-assemblages, such as the bivouacs built by army ants and the festoons made by swarming honeybees, mechanical stresses are expected to play an instrumental role in determining the final shape of the structure [89]. Indeed, as these self-assemblages grow in size with the addition of workers over time, so does their total weight. In order to maintain their structural integrity as they grow, they must, therefore, adopt shapes that help distribute the weight of the structure over its different attachment points and over the individuals that it is composed of. There is preliminary evidence for the influence of this weight distribution problem [92], but how it translates from the behaviour of the individual workers remains to be determined.

Collective development is also strongly influenced by environmental factors. For example, the structure of *Eciton* army ants bivouacs changes over the course of a bivouac's lifespan in order to regulate microclimatic conditions [93–96]. Bivouac shape is also influenced by seasonal fluctuations and local topology [97]. Like *Eciton*, *Oecophylla* weaver ants also form both worker chains [98] and non-subterranean nests, specifically arboreal leaf pouches glued shut with the silk of their larvae [99]. Weaver ant chains seemingly change in length over time based on cues from the number of ants already in the chain and in response to environmental attractants such as visual stimuli of interest to the ants [98].

However, the influence of environmental factors on the morphology of collective architecture is not well understood. In particular, the precise relationships between environmental inputs, individual behaviours and features of the collective architecture are not known. Unlike the information available on the migratory paths and ultimate fate of individual cells and cell types during embryo development [100,101], researchers in the field lack tools to precisely trace the movements of the individual workers comprising an army ant bivouac or a honeybee festoon, for instance. This is made particularly difficult by the three-dimensional nature of such structures which results in visual occlusions and requires the use of imaging techniques such as computed tomography (CT) that present many technical challenges when imaging a large number of live animals.

However, some progress has been made using smaller structures that are more amenable to the observation of the behaviour of individual agents. For instance, studies of bridges and scaffolds built by army ants showed that individual ants can adjust their behaviour dynamically to local variations in traffic and substrate during the construction process [69,102]. Similarly, the relatively low number of individuals comprising blackworm blobs allows for individual tracking and for observing the development of the collective structure over time [61]. After these worms aggregate into blobs, a division of labour allowing for directional travel is established through a combination of pulling and wiggling motions from different worms, with worms occupying different positions in the blob being more or less rigid (figure 3*b*) [61]. Capturing more detailed time series data on formations such as these would enable the identification of mature and immature states and allow for predicting the success or failure of the developmental trajectory of specific collective behaviours and of the entire group itself.

### (c) Maturity

Maturity is characterized by a disinvestment in growth processes with survival priorities being maintained. In the case of most unitary organisms, this stage includes reproduction, though by contrast many collective structures do not reproduce. Once at maturity, all biological structures maintain their functioning over a generally wide range of conditions that can fluctuate dramatically over time. This ability to maintain homeostasis once maturity is reached is critical to the fitness of the organism that harbours these structures [103–105]. This logic also applies to the superorganismal level, which requires that its collective organization be functionally stable in a sometimes rapidly changing environment.

The study of homeostatic circuits has a long history in animal and human physiology and neurobiology in particular. Indeed, many diseases are caused by deregulations of these circuits, either by external agents [106–108] or internal disorders [109,110], putting a spotlight on the mechanisms by which organisms maintain their functioning in normal and adverse conditions.

In mature collective systems, however, the study of homeostatic mechanisms remains sparse and, for the most part, theoretical. Moreover, such studies are largely focused on the robustness of collective behaviours in response to ‘noise’ in environmental conditions or on the individual behaviour of the members of a group [111–113]. However, robustness to perturbation is only one aspect of homeostatic balance and more complex mechanisms of regulation, including mechanisms for restoring homeostasis or for re-establishing function during chronic disequilibrium, are largely understudied in collective systems.

Superorganisms attempting to maintain homeostasis do not exist in a vacuum. Instead, they function as phylogenetically diverse ecosystems, mirroring the species-rich association between multicellular organisms and their microbiome. In the same way that interactions between microbial organisms and their multicellular hosts may be either mutually beneficial or antagonistic, army ant colonies constantly interact with a wide diversity of species [114,115], from microbes to birds, and these relationships range from commensal invertebrates feeding on colony waste [114,115], to symbiotic beetles that travel with the colony [116], to parasitic antbirds [117]. In this context, a colony's ability to maintain its health over time may rely on more than the actions of its conspecific members, highlighting the importance of studying the influence of a colony's micro/macrobiome on its collective behaviour.

**Figure 4.**
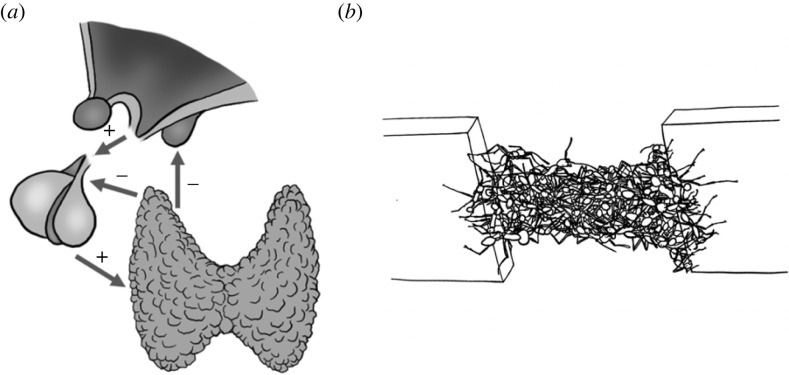
The hypothalamic pituitary thyroid axis ((*a*); [120]) and army ant bridge hysteresis ((*b*); [121]) as examples of processes of homeostasis during maturity.

Understanding homeostasis in single individuals has significantly progressed as a result of targeted and hypothesis-driven experimental perturbations [118,119], as in the case, for example, of the hypothalamic pituitary thyroid axis (figure 4*a*) [120]. In the study of animal collectives, it is typical to observe a given collective behaviour in multiple contexts to understand its driving factors. However, perturbations—that is, modifications of the context during the course of an experimental trial—are less commonly performed. Yet, such manipulations are key to understanding homeostatic mechanisms and their limitations.

Notable exceptions include studies of collective decision-making in honeybees and ants in which the location, quality and/or access route to resources are modified after a colony performs an initial collective choice [20,122–126]. These experiments revealed behavioural mechanisms (e.g. cross-inhibition) allowing colonies to avoid deadlocks and quickly abandon resources when they are no longer profitable.

This general approach was also recently used to determine how army ant self-assembled bridges maintain their function despite variation in their usage rate and in the structure of the substrate they are built on. Army ant bridges result from ants linking their bodies together [102] to span gaps along their foraging and migrating trails. They are formed in response to constraints on movement, traffic rates and substrate properties [102], and they remain functional even when these inputs are varying dynamically over time. In particular, it has been shown that ants forming the structure of a bridge respond with a small time delay to changes in the traffic intensity over it [68]. This allows the collective structure to ‘ignore’ fast variations in its usage while keeping it responsive to longer-term changes [68]. Similarly, the ants forming the bridge respond differently to a shortage of workers in the structure than to an excess of it. This creates a hysteretic response of the bridge that constructs and deconstructs itself at different rates when the gap it spans increases or decreases in size over time (e.g. when built between two leaves that can move in the wind; [121]). As a result, the bridge retains its integrity and function more efficiently than if the behaviour of the ants was impervious to these changes (figure 4*b*) [121]. Recent efforts to model these rules have recently served as inspiration for designing controllers for cooperative robots [127], although so far only in two dimensions.

Unlike army ant bridges, super-organismic colonies do engage in reproduction during maturity, with most species producing reproductive brood only when a colony has reached a sufficient size or age [128,129]. Efficient temperature and humidity regulation are critical to the growth and development of social insect brood, as well as to general colony health. As a result, many social insect collective behaviours have evolved for maintaining the homeostasis of the atmospheric conditions of the colony. For instance, colony-wide behavioural states in honeybees can be accurately predicted by observing changes in temperature [130], highlighting the general importance for bees to manage colony temperature, both inside the nest [131] and when swarming occurs [132]. This thermostasis is achieved through a variety of cooling and heating behaviours by individual workers, both at small and large scales [133]. The heat generated by worker bees is also used to kill intruders [134], much in the way that fevers in multicellular organisms are used to kill pathogens, participating further in the global homeostasis of the colony.

Atmospheric homeostasis can also be achieved through modification of the environment, for instance by constructing efficient gas exchange structures within a nest as can be found in leaf-cutting ants [135] and mound-building termites [136]. Experiments have demonstrated that these structures are built by the accretion of soil material along pre-existing air currents and that they are continuously modified and repaired throughout the life of the colony in order to ensure sufficient air renewal and stable atmospheric conditions within the nest [137–139]. This behaviour has sometimes been compared to processes of continuous tissue construction and repair within a multicellular organism and can also be abstracted to solving problems in urban planning [140]. While such nest architecture regulation occurs on a longer timescale than that of the more transient collective behaviours described previously, it nonetheless reflects the importance of understanding the successive stages of the collective behaviour of a colony over its entire lifespan.

Finally, studying homeostatic mechanisms promotes a more integrative approach to understanding the maturity of collective systems. Indeed, it requires determining how the states of the environment and of the collective are perceived by individuals, how that perception is modulated by innate and/or acquired inter-individual differences, and how it translates into individual behavioural responses that, ultimately, maintain (or return) the collective near its homeostatic state.

Examples of this integrative approach to understanding collective homeostasis include recent studies using clustered regularly interspaced short palindromic repeats/CRISPR-associated protein 9 to knock out odorant receptors in clonal raider ants [141,142]. A single genetic change led to attenuated aggregation and trail-following behaviours in affected individuals, emphasizing the extreme importance of robust perception and communication processes—here via chemical signals—for maintaining a group's integrity and functioning [141,142].

### (d) Dissolution

As discussed in the previous sections, a fair amount of information is already known, albeit in a disconnected fashion, about the formation, development and maturity of several types of collective behaviours. In comparison, the causes and mechanisms of the dissolution of collective formations are rarely characterized or even acknowledged in the literature. Yet, senescence and death can have adaptive value [143–145] and, therefore, it is relevant to understand the conditions that trigger or influence these processes in collective systems, as much as in unitary organisms.

**Figure 5.**
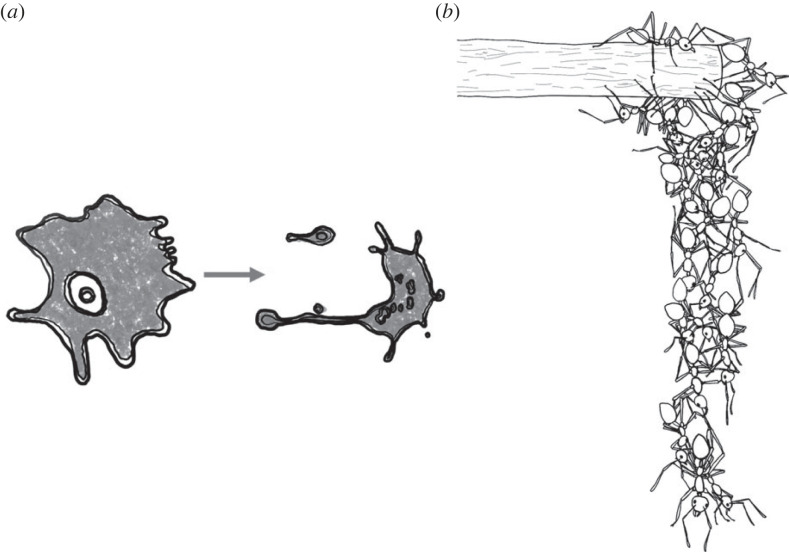
Apoptosis ((*a*); [13,151]) and weaver ant chain disassembly ((*b*); [98]) as examples of senescence/dissolution.

Here, maybe more than in reference to previous life stages, it is important to distinguish between the timescale of a group's lifetime and that of the transient collective behaviours it may exhibit. The former concerns the dissolution of the group itself and the cessation of all its activities. In this context, it adheres to the traditional meanings associated with senescence and death in unitary organisms. In many social insect species, the natural senescence and death of the entire colony closely follow that of the queen in cases where she is the sole reproductive individual [146–148]. However, in colonies where new reproductive individuals can emerge and replace the old ones, the mechanisms of colony senescence are not well known and colony death may be solely caused by externalities such as resource exhaustion or catastrophic events [149,150]. A similar observation can be made for groups of unrelated individuals (e.g. flocks of birds, schools of fish, etc.) that can, theoretically, ‘live’ forever as long as resources and other environmental conditions are suitable for their members to reproduce. In this context, it may be worth considering whether such groups are capable of decaying and disappearing spontaneously, in the same way that unitary organisms eventually die despite favourable environmental conditions (figure 5*a*). The answer to this question may reside in the ability of the group to limit the expression of selfish behaviours by its members, for instance.

The timescale of transient social behaviours instead concludes when a given collective action of a group ceases, though groups retain the ability to perform the action again in the future. In this case, the terms ‘senescence’ and ‘death’ may be better understood as ‘deterioration’ and ‘cessation’. These steps are rarely considered in collective behaviour studies, at least not explicitly. They can, however, have a significant impact on the global output of the group. For instance, army ants present in the self-assembled bridges and scaffolds built along foraging and migratory trails spontaneously abandon these structures when they perceive a significant drop in the traffic they support [69,102,121]. As a result, the support structures disassemble themselves when no longer useful. Since an estimated 2–20% of a colony's workforce can be engaged in their construction/maintenance at any one time [102,152], a usage-based behavioural mechanism allowing the interruption of collective construction processes probably leads to significant energy savings and/or better work allocation at the colony level. Similarly, hanging chains formed by weaver ants spontaneously disperse themselves when visual attractants are removed or when chains become overly long without finding footing, reducing the time collectively lost by the workers forming these structures (figure 5*b*) [98]. Finally, ants that are denied access to a food source at the end of a foraging trail will halt their recruitment [153]. They will even lay a ‘stop’ pheromone that discourages other ants from following an attractive trail towards an exhausted or overcrowded food source [154]. As a result, the traffic along the trail may progressively disappear, allowing the colony to save its workforce's energy or redirect it towards alternative sources.

The processes that lead to the dissolution of groups and the cessation of their collective behaviours are potential targets for natural selection. As such, they should be more prominently featured in studies of collective and social behaviours. Moreover, understanding the specific factors that induce and modulate collective dissolution may have very practical implications. For example, parasite infections in locusts can disrupt the gut bacteria responsible for producing pheromones leading to swarming behaviour [155]. This can interrupt locust gregarization and may be useful in limiting the devastating crop loss associated with these insects. Finally, several collective behaviours in humans result in negative outcomes such as costly traffic congestion [156,157], deadly stampedes or episodes of mass violence [158,159]. Better knowledge of mechanisms for collective dissolution may help mitigate these phenomena through new paradigms for urban design and crowd management, for instance.

## 3. Self-assemblages as model systems for studying collective ontogeny

While an ontogenetic approach offers benefits for understanding all forms of collective behaviours, it may be more immediately applicable to certain collective systems. This is the case for the self-assemblages built by various species of social insects. Indeed, these dynamic, collectively built structures possess many features reminiscent of developmental processes in multicellular organisms [39]. For instance, they mirror the architectural properties of embryos, in that both types of objects grow in three dimensions over time by the addition of seemingly identical units (cells in one case, workers in the other) and are influenced by internal forces directing both their overall shape and the sorting and positioning of the units within [13,88,160]. Additionally, super-organismic self-assemblages, like embryogenesis, lead to the emergence of heterogeneous structures whose different subparts serve each specific function necessary for the success of the whole. Finally, as alluded to in the previous section, information about stages of ontogeny is rapidly becoming available in the context of social insect self-assemblages and a number of recent studies have started investigating their relationships in a somewhat ontogenetic approach.

The correspondence between super-organismic self-assemblage and embryogenesis is, of course, not exact. Self-assemblages differ from embryonic development primarily in terms of timescale. The transitory social groups involved in feats of self-assemblages have short lifespans, on the order of minutes or hours, compared to the length of time occupied by embryonic processes for multicellular organisms. Other forms of collective architecture, however, such as the nests created by social insects, operate on a longer timescale more comparable to embryogenesis. Despite being non-living structures, unlike self-assemblages, such nests are shaped by the ongoing collective behaviour of their builders over the lifespan of the colony and reflect changes in its life stages. In addition, the movement of a cell is significantly constrained over its lifespan compared to the positional flexibility of individual workers in an army ant bivouac or a honeybee cluster. Cell function is also strongly linked to physiological location [161,162] and cells stay largely in one place, or, in the case of cells in circulatory systems, inside one organ system until they die. Self-assemblages, however, frequently disassemble and reform and are characterized by a more dynamic organization [93]. Because of this, precise morphology at various points in development is not well characterized for many forms of collective architecture. Such differences should be considered when drawing upon studies of embryo development for application to self-assemblages. However, this does not preclude the application of a wealth of ontogenetic tools and concepts to this type of behaviour.

### (a) Honeybee clusters and army ant bivouacs

Out of the numerous forms of collective architecture exhibited by social insects, two, in particular, are promising model systems for studying collective ontogeny: honeybee clusters and army ant bivouacs. They both are large-scale objects (around 10–80 000 individuals in a honeybee cluster [163]; up to one million in an army ant bivouac [164]) that are big enough for their constitutive units to experience vastly different local conditions (e.g. differences in temperature and mechanical stress between the surface and the core) and, therefore, create heterogeneities in the global structure. These structures self-assemble fast enough that this process can be experimentally observed multiple times a day and progressively enough that it is possible to characterize their construction dynamics in detail. Recent studies have shown that they can be observed in three dimensions and experimentally manipulated in laboratory and semi-field conditions [92,165]. Finally, they have a clear beginning and end (these structures spontaneously assemble and disassemble throughout the day in natural conditions), which allows for the study of their complete ontogenetic arc.

Honeybee collective architecture composed of living workers includes festoons and clusters or swarms and these structures have clear developmental patterns. Festoons typically emerge in the spring prior to the construction of new combs [166], although what triggers their formation is debated [62]. Festoons are built from chains of linked bees, and the distance these chains span across a gap may allow bees to make measurements to determine the position of a new comb [32]. Such measurements are important for preserving ‘bee space’, the gap between adjacent combs allowing workers to move freely [63,167]. The shape of a festoon often grows to resemble the shape of the comb eventually constructed in the area in question [32] and it disassembles as a new comb fills in the space [168]. Honeybees swarm most commonly as part of colony reproduction or splitting, a process often initiated when a colony outgrows its hive, causing crowding and the dilution of the queen's pheromone signal [169]. Colonies reproduce through the founding of new nests by these departing swarms in company with a queen [170]. When such swarms rest during their search for new nest sites, the group forms an outer ‘mantle’ layer composed of carefully aligned older workers, with younger workers inside the cluster [171]. This mantle differs in structure depending on weather conditions [171]. Swarms dissolve and transition to nest construction after scouts identify a suitable location and recruit others [172–175], a democratic decision process [176].

The bivouacs of *Eciton* army ants are, by far, the largest of the diverse functional self-assemblages built by these ant species [32]. The bridges, scaffolds and ladders that help facilitate the traffic along their busy exploratory, raiding and migratory trails are rarely composed of more than a couple of hundreds of individuals each [69,102,121]. In comparison, bivouacs can be made of hundreds of thousands of interlocked workers [165]. Their structure is also highly heterogeneous, with a dense outer shell surrounding a looser core containing empty spaces for storing and protecting the colony's queen and brood from outside elements [165]. Bivouacs are also very adaptable constructions. For example, they can ‘live’ for a few hours only when the colony is in its migratory phase and moves to new hunting grounds every night in order to feed their numerous hungry larvae; they can also last for weeks at the same location when the colony is in its stationary phase and contains no larvae that need feeding [177]. They can be built in a variety of environments, to which they adapt their shape accordingly [97], and there is strong evidence that their structure dynamically adjusts itself to climatic changes in order to thermoregulate [67,95]. Finally, the construction process of army ant bivouacs is progressive, starting with the formation of hanging chains that gradually coalesce into a usually cone-shaped structure, though the final shape may depend on the shape of the building substrate [97]; the bivouac then grows by the addition of workers on its outer layer while empty spaces appear little by little within the core as part of the ants inside detach themselves from the structure [165].

### (b) Taking inspiration from embryology to modernize the study of self-assemblages…

Distinguishing between the proximate and ultimate causes of mechanical self-assemblages is a challenging task. Indeed, many construction mechanisms identified in collective architecture may be analogous to the ‘spandrels of San Marco’—that is, indirect products of selection for other functions as defined by Gould & Lewontin [178]. It is unknown for instance, whether the hollow pockets formed inside a bivouac of army ants [165] have evolved for safely storing the queen and its brood, or whether they were rendered necessary for structural reasons, such as better distribution of the weight throughout the structure. Similarly, the thicker outer layer of interwoven workers may have evolved for increasing the impermeability of the bivouac to rainwater, but it could as well be simply a byproduct of the progressive hollowing of the core during the growth of the structure.

In this context, Tinbergen's ontogenetic angle, applied to self-assemblages, offers a relatively untapped approach to these questions. For instance, if mechanical constraints are the primary drivers behind the evolution of the bivouac structure, then changes in the distribution of ants, the size and number of empty spaces and various other metrics about the shape and organization of the bivouac as it grows over time should be mostly predictable from physical principles. Deviations from these principles would indicate that biological factors also play a significant role.

However, while self-assemblages in honeybees and army ants are promising model systems for studying collective ontogeny, several challenges limit our theoretical understanding and the possibility of making detailed observations of these structures over time. For example, tracking the identity of the individuals making up the different parts of a dense, three-dimensional self-assemblage is virtually impossible with current observation methodologies. Yet, this kind of detailed temporal information may be critical to characterizing the dynamical, behavioural processes from which the structural properties and the functional outcome of the self-assemblage emerge.

Technically, tracking large numbers of individuals within self-assemblages, swarms and other large groups has made enormous progress over the past 10 years, both in laboratory and field conditions [37]. This is in part owing to the greater availability of drones for aerial surveillance and remotely transmitting markers that can be attached to individuals [37], in particular allowing for tracking larger animals that traverse wide territories [179] or aquatic or flying species [180,181]. In parallel, new algorithms have been created to aid in the automatic detection of individuals in groups on video, and in determining body postures characterizing specific behaviours [37]. Increasingly sophisticated machine learning techniques have been developed to automatically categorize behaviour [182–187] in an ever-expanding number of species [188]. Despite these incredible advances, many of these new methods are limited to tracking individuals in two dimensions, and those that work in three dimensions can only be applied to loose swarms and not mechanically connected groups like honeybee clusters and army ant bivouacs.

Taking inspiration from technologies employed in embryology to study mechanical self-assemblages has the potential to resolve some of these challenges. For instance, CT scanning has been used to study the location of cells in developing embryos in three dimensions [189], as have immunohistochemical techniques to mark and trace subsets of individual cells [190]. Recently, the internal three-dimensional structure of living bivouacs [165] and bee clusters [92] has been described using CT scanning at different time points during the construction process. With this data, it is now possible to characterize the morphological development of certain forms of collective architecture and to look for potentially conserved structural organizations across different assemblages and species, analogous to the concept of the *bauplan* in ontogeny [191]; a shared behavioural skeleton shaping collective morphology.

Given enough computing power, it is even possible to accurately reconstruct the position of every individual in the structure, provided that they were not moving during the scanning process. This last point, however, illustrates a bottleneck with CT scanning and similar technologies: current affordable solutions that are adapted for observing large self-assemblages have a poor temporal resolution, making it practically impossible to track individuals over time [92,165]. Importing marking methods from embryology may somewhat help circumvent these limitations. For instance, chemical and genetic tools have been used to identify key cell subpopulations in developing embryos and trace their functions over time [192,193]. While not yet employed in studies of self-assemblages, such labelling methods could facilitate more advanced forms of tracking of genetically or transcriptomically relevant subpopulations, especially if they can be combined with three-dimensional CT imaging. When viewing a collective formation as a superorganism, chemically tagging individuals on the basis of gene expression related to specific behaviours, particular genetic backgrounds (e.g. patriline), or simply as a marker of identity in species that are difficult to mark using traditional methods, could be useful in creating three-dimensional visualizations of movement and interactions over time.

Finally, progress in embryology and developmental biology has been largely driven by performing perturbation experiments, such as chemical disruption, ablation, transplantation and mechanical deformation [118–120]. Similar approaches have successfully been applied to studying the properties of loose swarms [91] but they remain to be applied systematically to mechanical self-assemblages. For instance, manipulating collective architectures by removing influential subpopulations or forcing mechanical stresses onto them would allow for assessing the physical and biological drivers of mechanical self-assembly.

### (c) …and their theoretical modelling

Computer and mathematical modelling have long been used to study the mechanisms and evolution of various forms of collective behaviour [194–197]. The existing models are highly diverse in both their complexity, level of description and method to compute their outcomes. For instance, some models will represent each individual as a discrete and unique autonomous agent—a microscopic perspective [198,199]—while others will approximate the entire population as a continuous time-varying density field—a macroscopic point of view [199,200]. In between, kinetic modelling offers a ‘mesoscopic’ view by representing the actions of many individuals as probability distributions [196,198,199]. Typically, more microscopic approaches more precisely describe the behaviour of each individual as well as the dynamics of the group but can be limited by prohibitively time-intensive computations and a lack of generalizability to other collective systems [201]. The inverse is true for macroscopic modelling approaches [201]. Mesoscopic approaches sit somewhere in between both in terms of computational complexity and general applicability [199,202]. Moreover, some models of collective behaviour will explicitly represent the spatial relationships between the members of a group, for instance, to understand how the collective dynamics affect and are affected by the distribution of individuals [203,204]. Others, on the contrary, will simplify the system solely to the interaction network between individuals and focus on the flow of information between them [205–207].

Existing models of collective animal behaviour are predominantly two-dimensional and they almost exclusively focus on representing social interactions and not physical ones [21,197,208]. Therefore, they may not be well adapted to modelling army ant bivouacs and honeybee clusters—three-dimensional aggregates whose dynamics are most certainly driven by both social and mechanical interactions. These constraints, however, have been central to the development of theoretical models of developing embryos and tissue morphogenesis [209,210]. Indeed, a cell's behaviour is directly affected both by signals (chemical and electrical) emitted by neighbouring cells and by mechanical forces exerted on it by the matrix and/or other cells it is often physically attached to [28,29,87,88]. As a result, computer and mathematical models in developmental biology almost always integrate these different sources of information and constraints, and a variety of tools and techniques already exist to accelerate their computation. For example, the complexities of optical organ formation have been modelled, both computationally and physically, by including considerations from mechanical feedback and Wnt signalling, allowing the testing of developmental hypotheses [211]. Cellular development in another organ system, the epidermis, has been modelled by incorporating molecular input in the form of Notch signalling and mechanical input in a computationally efficient manner based on graphics processing unit algorithms [212]. Plant morphogenesis is similarly influenced by these main elements and has been modelled with an emphasis on the unique role of turgor pressure in plant growth and its interactions with cell wall physics and gene networks [213]. Adapting theoretical approaches from developmental biology such as these to study the ontogeny of social insect mechanical self-assemblages would help simultaneously describe the social interactions and physical linkages formed by the animals and would help generate answers to questions about weight distribution and structure heterogeneity during the construction and life of these dynamic three-dimensional structures.

Moreover, theoretical knowledge of the mechanisms involved in feats of collective behaviour has, in turn, catalysed major advances in robotics [214–219]. Forging a better understanding of the general principles governing collective behaviour, through an ontogenetic lens from formation to dissolution, can only improve the ability of bioinspired robot swarms to autonomously assemble and disassemble, and to modify their collective forms to maintain function in the face of dynamical environmental challenges.

## 4. Conclusion

Social groups and their collective behaviours have beginnings and ends, and in between are rich developmental trajectories that remain largely unexplored, at least explicitly. Yet, the fields of developmental biology and embryology have long demonstrated the benefits of an ontogenetic viewpoint to better understand the functioning and evolution of unitary organisms, including their behaviour. Here, we argue for integrating this viewpoint more frequently and explicitly in studies of biological collectives in general, and of superorganisms in particular. To illustrate our point, we have highlighted several organizational similarities between developing multicellular organisms and the collective behaviours of social animals. In particular, they share an ability to create complex, functional and heterogeneous structures without centralized control and they undergo similar developmental phases over their lifetime.

Current approaches to studying group behaviours, such as improvements in aerial and remote monitoring of animal movement, the use of machine learning in automating behavioural categorization, and increasing sophistication in modelling group dynamics, offer exciting insight into collective intelligence feats. However, we believe that there is more to be learned about collective formations as developing superorganisms and, in particular, about the time-varying factors involved in their formation, the emergence and maintenance of their functions, and their dissolution.

In this context, the mechanical self-assemblages of certain social insect species can constitute good model systems to systematize the ontogenetic approach in the analysis of collective behaviour because of their striking resemblance to developing multicellular organisms. Indeed, studies in this area would greatly benefit from importing tools from embryology and developmental biology, more specifically three-dimensional dynamical imaging techniques, hypothesis-driven perturbation experiments and sociophysical modelling approaches. Rigorously applying these approaches over the developmental time of superorganisms and their behaviours would generate new insights into the plasticity of collective intelligence processes and their adaptability to environmental changes. It would also provide general principles for creating collective artificial systems capable of autonomous development for applications in collective robotics [220,221], distributed artificial intelligence [222,223] and regenerative medicine [214,224].

## Data Availability

This article has no additional data.
